# Prognostic value of the DNA integrity index in patients with malignant lung tumors

**DOI:** 10.18632/oncotarget.25086

**Published:** 2018-04-20

**Authors:** Dimple Y. Chudasama, Zeynep Aladag, Mayla I. Felicien, Marcia Hall, Julie Beeson, Nizar Asadi, Yori Gidron, Emmanouil Karteris, Vladimir B. Anikin

**Affiliations:** ^1^ Division of Thoracic Surgery, The Royal Brompton & Harefield NHS Foundation Trust, Harefield Hospital, London, UK; ^2^ Division of Biosciences, Brunel University London, London, UK; ^3^ Scalab, Lille University, Oncolille, Lille, France

**Keywords:** circulating tumour DNA, Alu repeats, DNA integrity index, malignant lung tumours, liquid biopsy

## Abstract

**Introduction:**

Lung cancer survival remains poor in the western world due to late presentation in most cases, leading to difficulty of treatment in these advanced and metastatic patients. Therefore, the development of a robust biomarker for prognosis and to monitor treatment response and relapse would be of great benefit. The use of Alu repeats and DNA Integrity Index has been shown to hold both diagnostic and prognostic value, and as it is obtained from the plasma of patients, it can serve as a non-invasive tool for routine monitoring. This study evaluates the efficiency of this technique in malignant lung cancer patients.

**Methods:**

Plasma samples were collected from 48 patients, consisting of 29 lung cancer patients and 19 non-cancer controls. Alu repeat ratio and confounders were measured.

**Results:**

Observations showed a higher Alu repeat ratio amongst the cancer group compared to controls (*p=0.035)*, mean Alu ratio 0.38 (range 0.01-0.93) and 0.22 (0.007-0.44) respectively, ROC curve analysis AUC 0.61 *(p=0.22)*. Analysis by staging was more promising, whereby a higher DNA Integrity Index was seen in advanced cases compared to both early stage and controls, *p<0.0001;* AUC: 0.92 *(P=0.0002)* and *p=0.0006,* AUC – 0.88 *(p=0.0007)* respectively, however no significant difference was observed in the early stage compared to controls. Short term survival data also showed a DNA Integrity Index of >0.5 to be associated with poorer overall survival *p=0.03*.

**Conclusion:**

The results of this study show a potential use of Alu repeats ratios for prognostic purposes in the advanced setting for lung cancer patients.

## INTRODUCTION

Lung cancer is still one of the biggest killers in the western world today, with incidence remaining high, and as many as 46,403 new cases reported in the UK in 2014 alone [1, 2]. It has been reported that between 30-50% of non-small cell lung cancer patients (NSCLC) will undergo recurrence [3], and variable response rates to chemotherapy drugs have hindered survival rates. Routine biomarker monitoring post-surgery could be of significant prognostic value, informing of possible relapse sooner, thus allowing rapid treatment. In addition, identifying such a prognostic biomarker could allow finding patients who benefit from therapies (i.e. radiotherapy, chemotherapy) as a means of monitoring and predicting response. With the emergence of blood based biomarkers, this is now becoming a possibility.

Given the current challenges in isolation and characterisation of CTCs for diagnostic or prognostic purposes, analysis of DNA isolated from peripheral blood might shed a light to tumour activity. More specifically, a portion of this DNA, known as cell-free DNA (cfDNA) circulating in plasma or serum can be derived from normal cells, including normal apoptotic white blood cells as well as cancer cells.

Circulating tumour DNA (ctDNA) is the portion of circulating free DNA specifically derived from cancer cells [4, 5]. Typically in healthy individuals apoptosis of cells occurs naturally, and DNA is released and uniformly truncated in small fragments of 185-200 bp [6]. However, tumour necrosis generates a spectrum of DNA fragments with different strand lengths typically >200bp, due to the pathological process of cell death consisting not only of apoptosis but also necrosis, autophagy and mitotic catastrophe [7]. Ineffective deoxyribonuclease activity is also reported to contribute to longer DNA fragments due to incomplete digestion of genomic DNA [8].

In addition to its potential role as a detection and prognostic method, ctDNA was also evaluated as a way of monitoring tumour progression and testing whether a patient's tumour would respond to targeted drug treatments. The percentage of ctDNA originating from tumour cells however, has been estimated to range from 10% to 90% of the total cell free DNA population, its applicability as a plasma biomarker may therefore depends on the type of disease [9].

Classified as Short Interspersed Elements (SINE*),* Alu is the most abundant mobile element in the human genome [10]. A full length can span approximately 300 base pairs (bp) in length, and include two tandem monomer units, separated by a poly “A” stretch [10]. Studies have shown the Alu-repeat measurements to adequately predict the disease in cases of colorectal, ovarian, breast and lung cancers, however use of Alu repeats was shown to be ineffective in pancreatic cancer [6–8, 11, 12].

To test this hypothesis in lung cancers, we conducted a case control study to measure using a real-time PCR-based assay, the Alu repeats in a total of 48 plasma samples. For this study we have used two distinct set of primers: One set of primers amplified shorter DNA fragments (115 bp in length; reflecting the total cfDNA); whereas the second primer set amplified only the longer DNA fragments (247 bp; representing the amount of DNA released from cancerous cells). DNA integrity index was calculated as the ratio of qPCR results with the 2 primer sets: Alu247/Alu115. Detection of these longer ctDNA fragments and quantification of their relative abundance in plasma compared to short cfDNA fragments, calculating a DNA Integrity Index has already been explored with promising results [13, 14].

To summarise, this study evaluates the use of Alu repeat and DNA integrity Index in lung cancer patients, and its ability to diagnose lung cancer in both early and advanced stages. The study wishes to assess whether a higher DNA integrity index is associated with more advanced lung cancer, hence allowing differential staging and prognostic prediction of patients based on their Alu repeat ratio.

## RESULTS

### DNA integrity index is of prognostic rather diagnostic value

**Figure 1:**
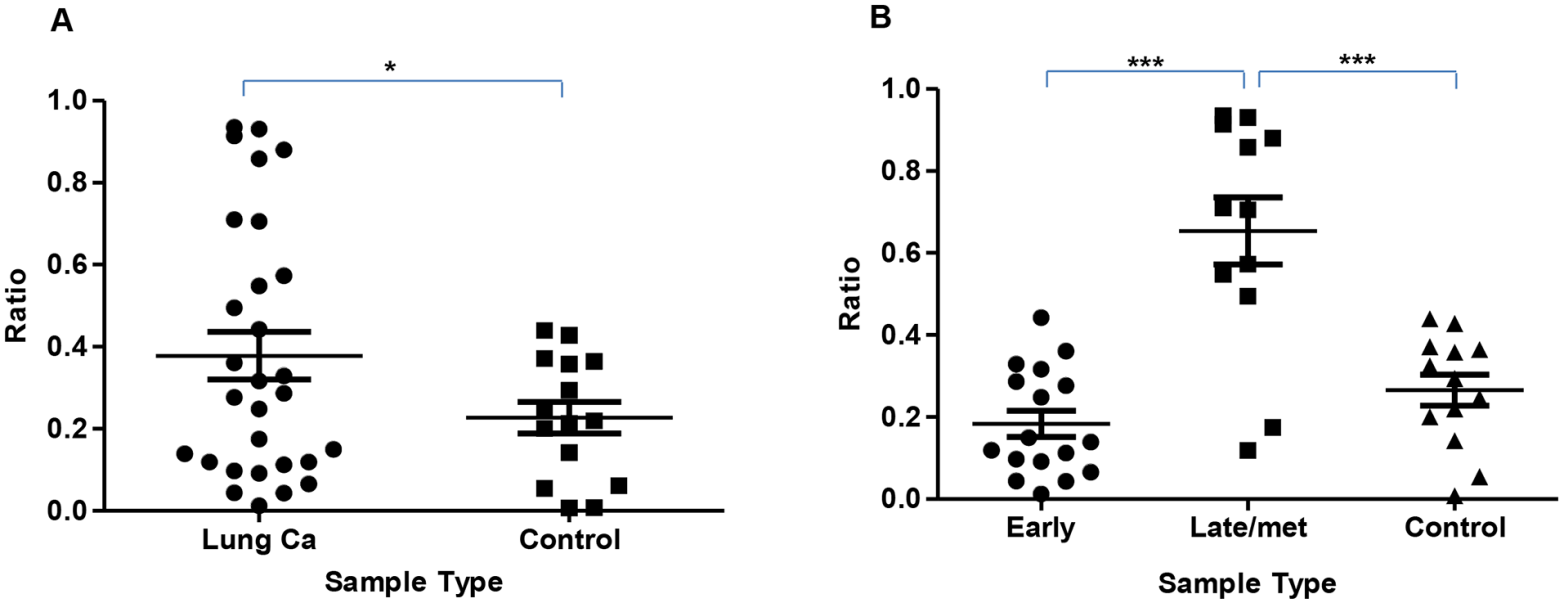
Scatter plot showing DNA integrity index **(A)** – shows a higher DNA Integrity Index in lung cancer compared to controls, *p=0.035*. **(B)** – shows an increased DNA Integrity Index in the advanced cancer group (III-IV including metastatic) compared to both early stage (I-II) and controls, *p<0.0001* and *p= 0.0006* respectively. Statistical significance was not achieved in the early stage vs control groups.

Plasma samples were collected and processed from 48 participants (Table 1), and extracted DNA processed by qPCR for Alu 115 and Alu 247. Data obtained from qPCR was analysed, and RQ values and DNA Integrity Index calculated and plotted (Figure 1). DNA Integrity Index is given as a value between 0-1 (raw data available in supplementary Table 1), with 1 indicating higher ratio of Alu 247, hence increased circulating tumour DNA burden [6, 7].

**Table 1:** Clinical details of recruited patients

Variable	Value	Percent (%)
Total	48	100%
Mean age (±SD)	60±15	-
Males/Females	25/23	52.1/47.9
**Pathology**		
All cancer	**29**	**60.4**
Primary lung cancer	22	75.9
Adenocarcinoma	15	68.2
Squamous cell carcinoma	7	31.8
Metastatic Cancer	7	24.1
Non-cancer control	**19^a^**	**40**
**Staging^b^**		
I-II	17	77.2
III-IV	5	22.7

^a^ Non- cancer controls include 2 patients awaiting bullectomy surgery.

A higher DNA Integrity Index is seen in the lung cancer group compared to controls, *p=0.035*. (Figure 1A). Comparison of the DNA Integrity Index in advanced cancer cases to both controls and early stage cancers are shown to be significantly higher, *p=0.0006* and *p<0.0001* respectively (Figure 1B).

Using a one-way ANOVA, comparing control, early and late cancer had a significant overall effect on Alu levels (F(2,45) = 26.00, p < 0.001). Following this, we conducted a contrast analysis and found that, both disease groups had significantly higher Alu levels than controls (t(45) = 3.46, *p = 0.001*). Controls had significantly lower Alu than late group patients (t(45) = 6.20, *p < 0.001*). Controls and early cancer groups were not different on Alu levels (t(45) = 0.69, p > 0.05), 4. Late group patients had significantly higher Alu levels than early group patients (t(45) = 6.67, *p < 0.001*).

**Figure 2:**
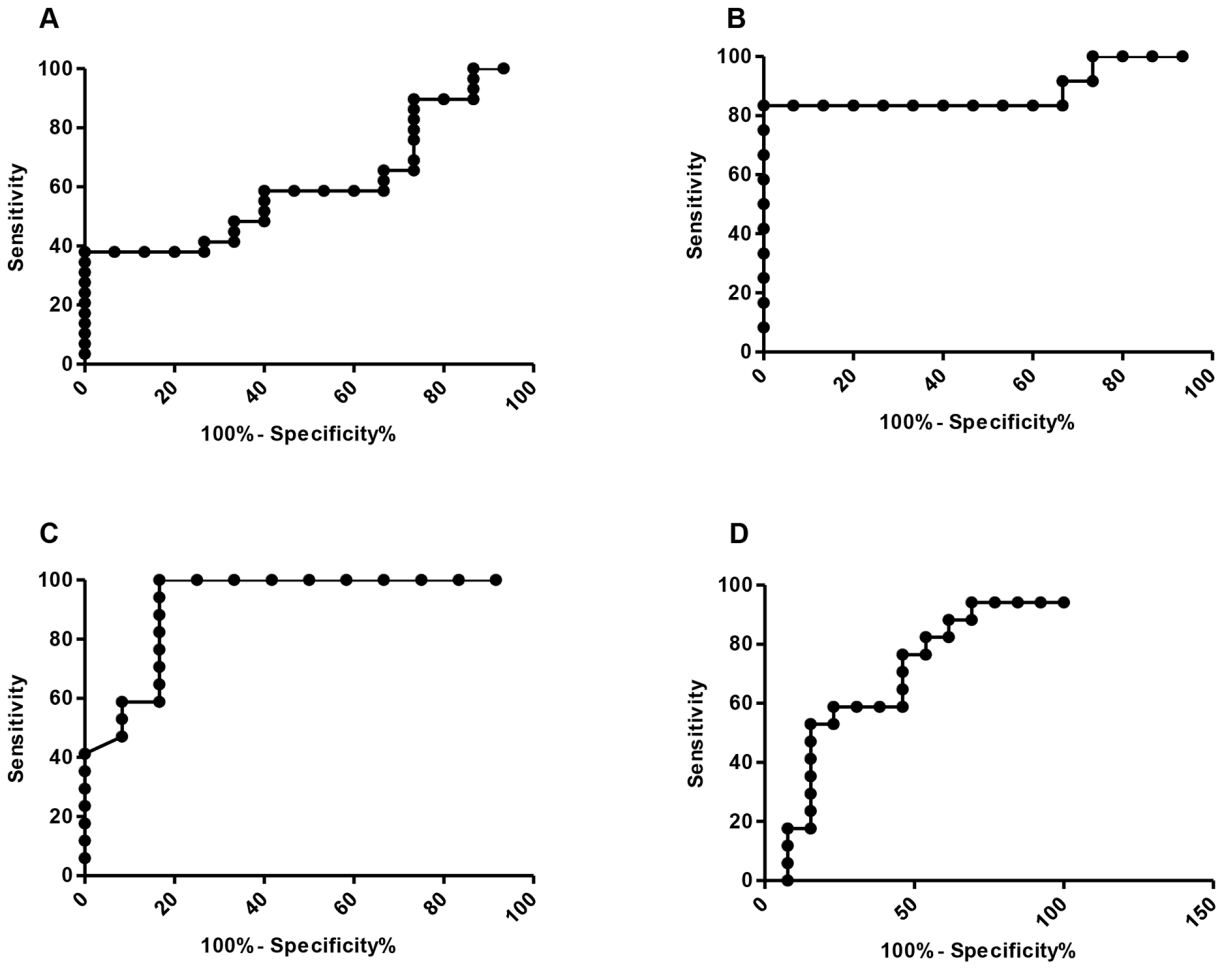
ROC curve analysis of diagnostic sensitivity and specificity of using ALU repeat ratios **(A)** – all lung cancer vs controls, AUC – 0.61, p = 0.22; **(B)** – advanced cases vs controls, AUC – 0.88, p=0.0077; **(C)** – Early stage vs controls, AUC 0.67, P=0.23; **(D)** – advanced cases vs early stage, AUC – 0.92, P=0.0002.

We expanded on these observations by calculating sensitivity and specificity data; obtained by receiver operative characteristics (ROC) curves, and calculation of area under the curve (AUC), using 95% confidence intervals (Figure 2). There was strong sensitivity and specificity in advanced cases to early, and advanced cases to controls, AUC – 0.92, *p=0.0002* and AUC – 0.88, *p=0.0077* respectively. Poorer sensitivity and specificity results were seen in the all lung cancer vs controls and early vs controls, AUC – 0.61, *p=0.22* and AUC – 0.67, *p=0.12* respectively.

Collectively these data, demonstrate that DNA integrity index is only useful when late stage is compared to early one, thus demonstrating a potential use of this test for prognostic purposes.

### DNA integrity index can determine survival

On comparison of pathology and staging, similar mean DNA Integrity Index were calculated in the early stage primary lung cancer group and controls, with considerable overlap as shown by the range 0.18 (0.01-0.44) and 0.22 (0.007-0.44) respectively. Notably higher DNA Integrity Indexes are seen in the late stage and metastatic groups, 0.61 (0.04-0.91) and 0.58 (0.17-0.9). Little difference is observed in median survival (survival in days from surgery), where in early stage median survival is calculated at 434 days post operation, this is slightly higher in the metastatic group at 454 days and 460 days in the late stage group (Table 2). Median survival data using the 0.5 index, showed a median survival in days in the <0.5 and >0.5 group of, 434 and 457 days post operatively.

**Table 2:** DNA integrity index by staging, including median survival times, show advanced cases and metastatic patients to have a higher Alu repeat ratios, compared to early stage and non-cancer controls, 0.86, 0.51, 0.18, and 0.22 respectively

Pathology	Patients (n)	Mean DNA Integrity Index (Range)	Median survival
**Primary lung cancer:**			
** Early stage (I-II)**	17	0.18 (0.01-0.44)	434
** Late stage (III-IV)**	5	0.86 (0.71-0.93)	460
**Metastatic**	7	0.51 (0.12-0.93)	454
**Non cancer controls**	19	0.22 (0.007-0.44)	-

Median survival times however do not seem to be significantly influenced by a higher DNA Integrity Index.

**Figure 3:**
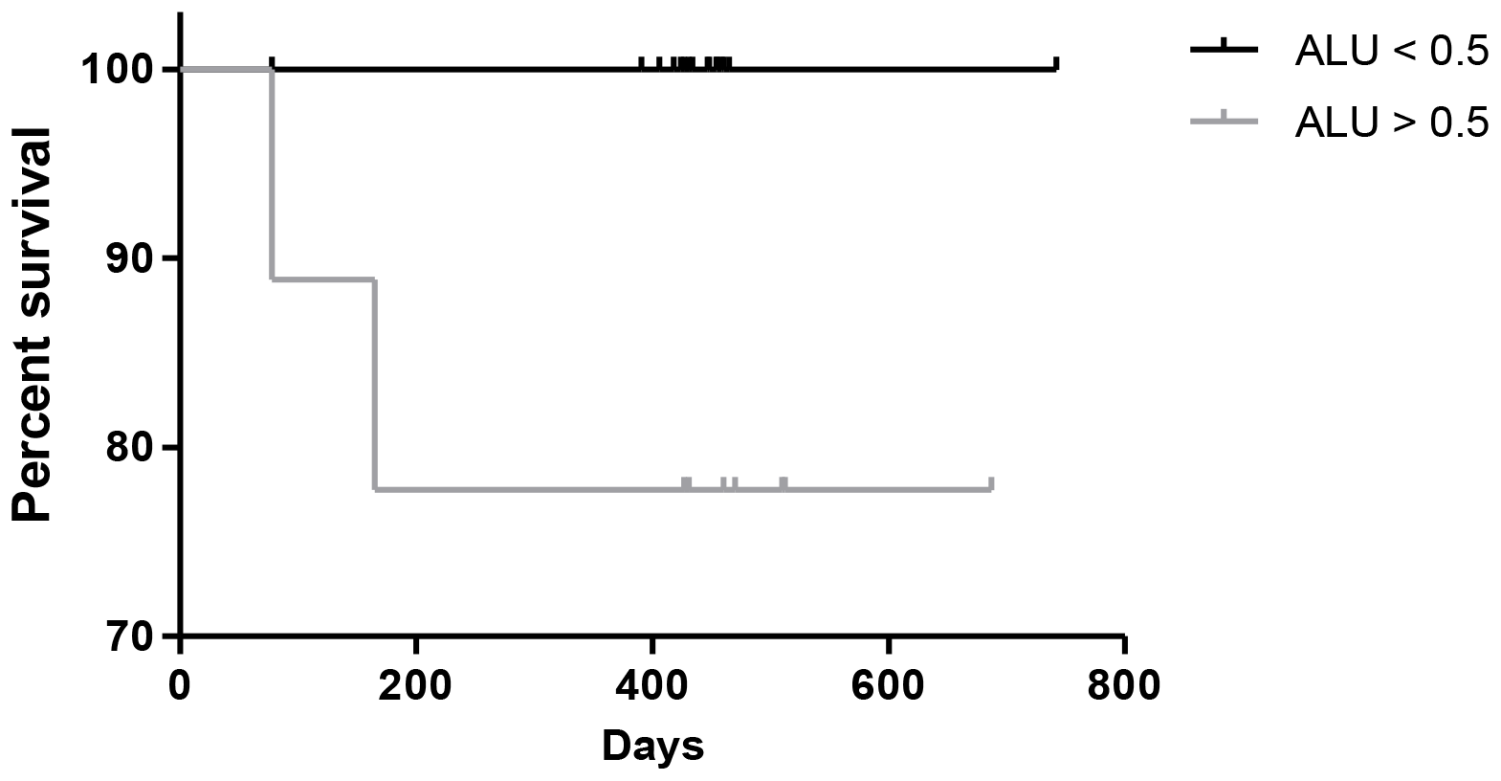
Kaplan Meier plot of overall survival in lung cancer patients based on DNA integrity index, patients with Alu reading >0.5 show poorer survival, p=0.03

Overall survival analysis by Kaplan Meier (estimates survival function, and proportion of patients alive over time) of the DNA Integrity Index, using the 0.5 index as a guide for high and low levels, showed significantly poorer overall survival in the patients with a DNA Integrity Index of >0.5, *p=0.03* (Figure 3). Demonstrating the prognostic value of DNA integrity measurements in advanced lung cancer cases.

### Sexual dimorphism and DNA integrity index

We further interrogated the data, using known factors for lung cancer that can influence survival. No significant correlations were found between Alu and CT tumour size (r = 0.21, *p > 0.05*; Supplementary Figure 1). Alu was also unrelated to age, smoking or diabetes mellitus (all *p > 0.05*). The only background variable associated with Alu was gender: women had significantly higher Alu (0.40) than men (0.15; t (27.33) = 4.09, *p < 0.001*. Subsequently, we found that the main group effect on Alu remained significant also after statistically controlling for gender (F(2,38) = 16.52, *p < 0.001*).

## DISCUSSION

Liquid biopsies are an attractive and more practical alternative for routine cancer monitoring, in contrast to current surgical biopsies. With ctDNA readily available in the plasma of cancer patients, various efforts have been made to exploit their clinical utility, one of which being the DNA Integrity Index [6, 8, 15]. This study evaluates the efficacy of the DNA Integrity Index, by means of Alu repeat analysis of ctDNA found in the plasma of patients with malignant lung tumours.

Based on the premise that tumour cells undergo more chaotic cell death compared to mostly apoptosis in normal cells, we would expect to find a higher ratio of fragmented DNA in the cancer cohort. As the disease progresses one would expect an increased DNA Integrity Index to reflect the increased tumour burden and thus shedding of fragmented DNA, potentially providing a means not only to identify and diagnose cancer patients, but also differentiate based on staging and advancement of disease. Umetami *et al* (2006) demonstrated a significantly increased DNA Integrity Index, even in patients with localised disease for colorectal cancer, demonstrating its utility as a diagnostic tool [6]. More recently, a study in 95 breast cancer patients, revealed a significantly higher DNA Integrity Index, compared to benign and control samples (*p<0.001)*. The group also reported sensitivity and specificity values of 85 and 100%, respectively, concluding the clinical utility of ctDNA, and correlation with TNM staging [16].

The results of this study show an increased DNA Integrity Index in the cancer cohort (average ratio: 0.38 (range 0.01-0.93) compared to controls (average ratio: 0.22 (0.007-0.44), *p= 0.035*. ROC curve (Figure 2A) analysis however indicates poor sensitivity and specificity. Furthermore, there is considerable overlap between the cancer and non-cancer cohort, which can also be seen in similar studies [6, 7]. No normal established baseline exists, healthy non-cancer patients will be expected to show both shorter and larger DNA fragments due to biological cell death processes. Inflammation and auto immune diseases are contributing factors to cell death rates, explaining higher DNA Integrity Index values in non-cancerous controls, with the lower ratio's seen in the cancer group attributed to effective DNA clearance, as well as minimal cell death [17]. Other factors such as trauma, stroke can also effect the Alu ratio, hence limiting the value for cancer [18–23]. This overlap between the cancer and non-cancer group, is suggestive of poor specificity and thus questioning its potential as a diagnostic tool.

However, when cancer patients are stratified by cancer stage (according to TNM staging system, as seen in Figure 1B, a significantly higher DNA Integrity Index is observed in the advanced patients (III-IV and metastatic), compared to both the early stage (*p<0.0001*) and normal cohort (*p=0.0006*). Moreover, ROC curve analysis demonstrates a high diagnostic accuracy between advanced cases and early stage and advanced cases and controls. The results of this study are generally in line with recent studies [6, 7], and suggests a prognostic role for this biomarker in advanced cancers. Literature also suggests that DNA released from malignant tumours into the bloodstream is enhanced by lymphovascular invasion, as direct lymphatic or blood flow through the tumours enables dissemination of viable tumour cells, and thus can enhance the diffusion of DNA released from dead tumour cells into the bloodstream. As a result, circulating DNA may be directly related to tumour progression and rate of tumour cell turnover, representing biologic tumour aggressiveness [7], which would agree with the results of this study.

Analysis of DNA Integrity Index against staging showed Alu repeat ratios of >0.5 in the advanced and metastatic patients (Table 2) when compared to controls and early stage. Supporting the notion that with disease progression more tumour DNA is shed into the circulation. Short term follow up data was also acquired, and overall survival plotted by DNA Integrity Index, whereby patients were divided by a ratio of either <0.5 or >0.5, based on the statistical data generated (Table 2). Furthermore, with the DNA Integrity Index falling between 0 and 1, the middle point of 0.5 was assumed a sensible cut off to distinguish higher ratios from lower. A similar approach and cut-off was also utilised in a recent study in breast cancers [16]. Overall survival was seen to be significantly worse in the >0.5 DNA Integrity Index cohort (*p=0.03*)., suggesting a direct association between the DNA Integrity Index and poorer prognosis, similar observations in overall survival were reported by Basnet *et al* (2016) in colorectal patients [24]. Moreover, the patients with a DNA Integrity Index of >0.5 were more advanced and metastatic cases, where a higher ctDNA content would be expected with cancer growth and spread, along with a poorer prognosis. Furthermore, these results suggest the utility of the DNA Integrity Index in predicting prognosis and OS.

Little difference was observed in the median survival in day's data, there was also no correlation seen between tumour mass and DNA Integrity Index, however one must consider the small number of advanced and metastatic patients.

Interestingly, ANOVA analysis revealed that women had significantly higher DNA Integrity Index (0.40) than men (0.15), *p < 0.001*. Subsequently, we found that the main group effect on the Alu ratio remained significant also after statistically controlling for gender (F(2,38) = 16.52, *p < 0.001*). This could be explained by sexual dimorphisms in DNA, in relation to DNA methylation and Alu repeats [25, 26].

Findings from our study are in line with similar studies, including Wang *et al,* (2003) who reported a significant increase in the DNA Integrity Index in ovarian and gynaecological cancers, concluding its clinical utility. Similarly studies in colorectal, periampullary, breast, ovarian, head and neck and prostate cancers [6–8, 27–29] also report positive results for the use of Alu repeats both diagnostically and prognostically. In contrast studies in pancreatic, gastrointestinal, colorectal cancer [30, 31] have concluded against their utility as a clinical tool due to poor sensitivity and specificity. The results from our findings support the latter, despite some promising results there is a lack of sensitivity and specificity in this method, highlighted by the overlap seen between the control and cancer cohorts. A larger sample size may alleviate some limitations from this study, however the DNA Integrity Index lacks value as a diagnostic tool, and requires improvements and standardisation of process. Positive results were obtained in the advanced stages, suggesting a potential role in patient staging, disease monitoring and prognosis.

## MATERIALS AND METHODS

In total 48 individuals were recruited to the study, including 29 lung cancer patients awaiting resection of their known primary or secondary lung cancer (Table 1). Samples were also taken from 17 healthy volunteers (males and females), and 2 patients undergoing non-cancer lung surgery. Prior consent was sought from patients following ethical approval from NRES (ref 14/LO/1284).

Ten ml blood samples were collected from patients into EDTA tubes, and centrifuged for 10 mins at 2500RPM. Two ml of the plasma layer was removed careful not to disturb the red blood cell sediment, the plasma was spun again for purity for a further 5 mins at 2500 RPM, and plasma extracted and stored at −80°C until further use. DNA extraction was carried out using the QIAmp DNA mini kit (QIAGEN, Germany) as per manufacturer's protocol. Extracted DNA was measured and stored at −20°C.

DNA samples were processed by quantitative PCR (qPCR) on the Quantistudio 7 (ABI), using SYBR green mastermix (ABI). Primer sequences were taken from Umetani *et al.* (2006), and obtained from Sigma, with the following sequence's Alu 115 forward: - 5’-CCTGAGGTCAGGAGTTCGAG-3’; reverse, 5’-CCCGA GTAGCTGGGATTACA-3’; Alu247 primers: forward, 5’-GTGGCTCACGCCTGTAATC-3’; reverse, 5’-CAGG CTGGAGTGCAGTGG-3’.

Relative quantification (RQ) was calculated for each sample, and DNA Integrity Index calculated as follows: RQ Alu 247/RQ Alu 115 [6].

Statistical analysis, all data's were analysed using the GraphPad Prism version 5. An F-test was performed to assess the variance s, and two-tailed unpaired Student's *t*-tests with Welch's correction for unequal variance were performed to assess significance. Receiver operating characteristics (ROC) curve and area under the curve (AUC) analysis were used to assess diagnostic utility. Survival data was calculated using Kaplan Meier plots. All statistics calculated with a 95% confidence interval.

## SUPPLEMENTARY MATERIALS FIGURES AND TABLES





## Abbreviations

(DNA): Deoxyribonucleic acid
(cfDNA): circulating free DNA
(ctDNA): circulating tumour DNA
(SINE): short interspersed elements
(ROC): receiver operating characteristics
(AUC): area under the curve
