# Supplementary material for: Prognostic value of the DNA integrity index in patients with malignant lung tumors
Source: Oncotarget. 2018 Apr 20;9(30):21281–8. doi: 10.18632/oncotarget.25086 (PMC5940399; doi:10.18632/oncotarget.25086)
Supplement: Supplementary file 1 [file oncotarget-09-21281-s001.pdf]

Prognostic value of the DNA integrity index in patients with malignant lung tumors

SUPPLEMENTARY MATERIALS

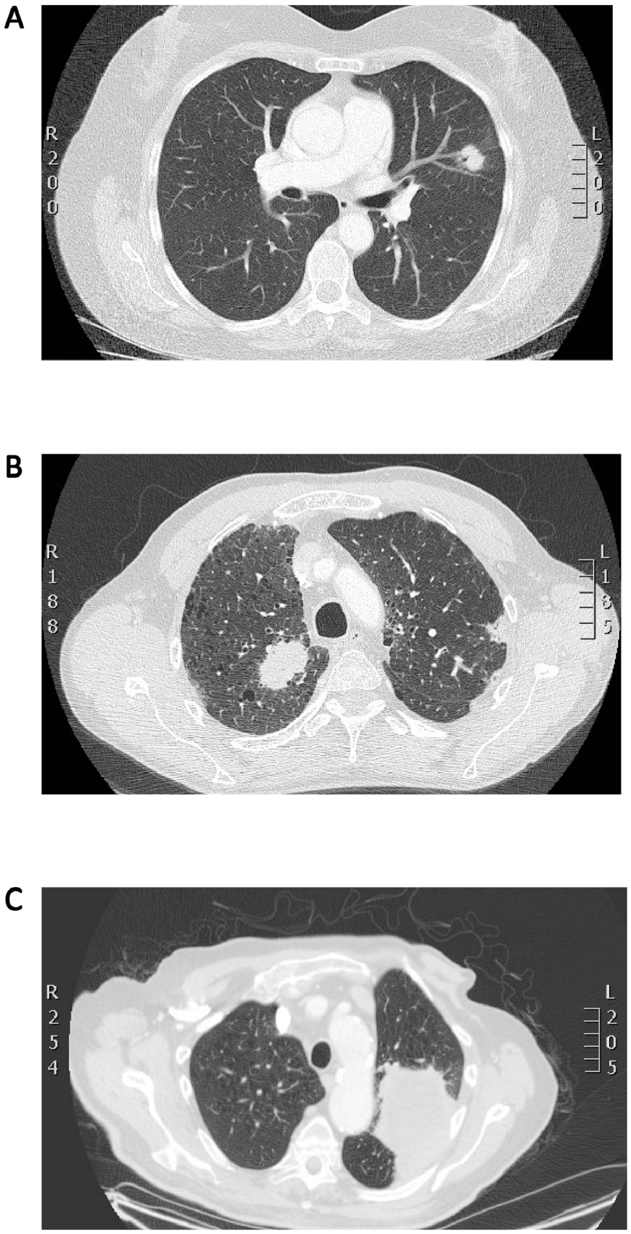

Supplementary Figure 1: CT imaging of lung cancer tumours. **(A)** Stage IA Non-mucinous Adenocarcinoma of the Lung; **(B)** Stage IIB Adenocarcinoma of the lung, and **(C)** Stage IV NSCLC.

Supplementary Table 1: Raw data file.

See Supplementary File 1
